# Effect of second timed appointments for non-attenders of breast cancer screening in England: a randomised controlled trial

**DOI:** 10.1016/S1470-2045(17)30340-6

**Published:** 2017-07

**Authors:** Prue C Allgood, Roberta Maroni, Sue Hudson, Judith Offman, Anne E Turnbull, Lesley Peacock, Jim Steel, Geraldine Kirby, Christine E Ingram, Julie Somers, Clare Fuller, Anthony G Threlfall, Rhian Gabe, Anthony J Maxwell, Julietta Patnick, Stephen W Duffy

**Affiliations:** a Centre for Cancer Prevention, Wolfson Institute of Preventive Medicine, Barts and The London School of Medicine and Dentistry, Queen Mary University of London, London, UK; b Peel and Schriek Consulting, London, UK; c Derby Teaching Hospitals NHS Foundation Trust, Royal Derby Hospital, Southern Derbyshire Breast Screening Service, Derby, UK; d Hull and East Yorkshire Hospitals NHS Trust, Castle Hill Hospital, Humberside Breast Screening Service, Cottingham, UK; e Plymouth Hospitals NHS Trust, Derriford Hospital, Primrose Breast Care Centre, Plymouth, UK; f South East London Breast Screening Programme, King's College Hospital NHS Foundation Trust, London, UK; g Sheffield Teaching Hospitals NHS Foundation Trust, Royal Hallamshire Hospital, Sheffield Breast Screening Unit, Sheffield, UK; h West of London Breast Screening Service, Imperial College Healthcare NHS Trust, Charing Cross Hospital, London, UK; i Theorize, Manchester, UK; j Department of Health Sciences and Hull York Medical School, University of York, York, UK; k Division of Informatics, Imaging and Data Sciences, School of Health Sciences, Faculty of Biology, Medicine and Health, University of Manchester, Manchester, UK; l Nightingale Centre, University Hospital of South Manchester, Manchester, UK; m Cancer Epidemiology Unit, University of Oxford, Oxford, UK

## Abstract

**Background:**

In England, participation in breast cancer screening has been decreasing in the past 10 years, approaching the national minimum standard of 70%. Interventions aimed at improving participation need to be investigated and put into practice to stop this downward trend. We assessed the effect on participation of sending invitations for breast screening with a timed appointment to women who did not attend their first offered appointment within the NHS Breast Screening Programme (NHSBSP).

**Methods:**

In this open, randomised controlled trial, women in six centres in the NHSBSP in England who were invited for routine breast cancer screening were randomly assigned (1:1) to receive an invitation to a second appointment with fixed date and time (intervention) or an invitation letter with a telephone number to call to book their new screening appointment (control) in the event of non-attendance at the first offered appointment. Randomisation was by SX number, a sequential unique identifier of each woman within the NHSBSP, and at the beginning of the study a coin toss decided whether women with odd or even SX numbers would be allocated to the intervention group. Women aged 50–70 years who did not attend their first offered appointment were eligible for the analysis. The primary endpoint was participation (ie, attendance at breast cancer screening) within 90 days of the date of the first offered appointment; we used Poisson regression to compare the proportion of women who participated in screening in the study groups. All analyses were by intention to treat. This trial is registered with Barts Health, number 009304QM.

**Findings:**

We obtained 33 146 records of women invited for breast cancer screening at the six centres between June 2, 2014, and Sept 30, 2015, who did not attend their first offered appointment. 26 054 women were eligible for this analysis (12 807 in the intervention group and 13 247 in the control group). Participation within 90 days of the first offered appointment was significantly higher in the intervention group (2861 [22%] of 12 807) than in the control group (1632 [12%] of 13 247); relative risk of participation 1·81 (95% CI 1·70–1·93; p<0·0001).

**Interpretation:**

These findings show that a policy of second appointments with fixed date and time for non-attenders of breast screening is effective in improving participation. This strategy can be easily implemented by the screening sites and, if combined with simple interventions, could further increase participation and ensure an upward shift in the participation trend nationally. Whether the policy should vary by time since last attended screen will have to be considered.

**Funding:**

National Health Service Cancer Screening Programmes and Department of Health Policy Research Programme.

## Introduction

An important indicator of the public health impact of the National Health Service Breast Screening Programme (NHSBSP) in the UK is the participation rate, defined as the percentage of women invited for screening who are screened adequately within 180 days of invitation (usually referred to as uptake in official reports). In England, participation following routine invitation fell from 74·4% in 2004–05 to 71·3% in 2014–15,^1^ and we are seeing a decline for the fourth consecutive year in a row, approaching the national minimum standard of 70%. In particular, participation among women invited for their prevalent (first) round of screening has decreased by an even greater amount (from 70·1% in 2004–05 to 63·3% in 2014–15).^1^ Participation in breast cancer screening also tends to be lower in areas of socioeconomic deprivation than in wealthier areas.^2^, ^3^

The NHSBSP invites women aged 50–70 years to mammographic screening every 3 years. The invitation letter includes a screening appointment with a given date, time, and place. An age eligibility extension to invite women aged between 47 years and 73 years is currently being trialled.^4^ The usual practice for non-attenders of the first offered appointment is to send them a second invitation letter, which can vary: some centres supply open invitations, asking women to telephone to make an alternative appointment; whereas others routinely offer second timed appointments, with date, time, and place stipulated. The Department of Health has advised NHS England that the approach with second timed appointments should be used.^5^ Second timed appointments are NHSBSP policy, although this approach is not universally followed.

**Research in context**

**Evidence before this study**

We searched the PubMed database with the keywords “breast cancer”, “breast screening”, “appointment”, and “non-attenders” for articles in English published between Jan 1, 1990, and Dec 31, 2016. This search retrieved eight papers, of which six were not considered relevant after title or abstract review. In 1998, Stead and colleagues compared second appointments with fixed date and time versus open second invitations for non-attenders of breast cancer screening in a randomised controlled trial in one breast screening centre in England, finding an increased participation rate with second timed appointments. Although the quality of the trial was good, its findings might not apply to current practice and target populations for screening since these have changed in terms of age and might have changed in terms of social support and employment status. In 2016, Hudson and colleagues reported the results of an observational study in north London comparing timed and non-timed second appointments, showing increased participation with timed appointments. We identified no trials of second timed appointments for non-attenders from outside the UK breast screening programme.

**Added value of this study**

Our randomised controlled trial assessed the effects of sending invitations for breast cancer screening with a second timed appointment to women who did not attend their first offered appointment. Unlike previous studies on this subject, the trial was done at a national level (six centres across England) in 2014–15. We could therefore analyse the efficacy of the intervention depending on a woman's location and level of socioeconomic deprivation since the sites in the study covered a wide range of socioeconomic status levels.

**Implications of all the available evidence**

Our findings show the positive effects of second timed appointments on attendance for breast cancer screenings. The results are of policy interest for early detection of breast cancer, because a simple change in the procedure of addressing non-attenders of breast cancer screening invitations could result in more women being screened.

Although some women might not attend their screening appointment because they have made an informed choice not to do so, some of them will not attend for other reasons. These women might find a second timed appointment more beneficial than an open invitation because it does not require any effort to book a new appointment with the screening centre. Previous findings suggest that participation is greater when a second timed appointment is given to non-attenders,^6^, ^7^ but further investigations are needed to identify the women who would be most and least likely to respond to the second invitation. For example, someone who has not attended their last three screening appointments might not attend whatever the form of the second invitation.

In a randomised trial published in 1998, Stead and colleagues^6^ found that the effect of second timed appointments declined with increasing time since last screen, and in a more recent observational study, Hudson and colleagues^7^ noted the same association, at least in absolute terms, in north London. The efficacy of this approach has not been investigated in a randomised trial, or quantified with precision, in the current target population for screening. Therefore, we did a randomised trial of second timed appointments versus open invitations for non-attenders within the NHSBSP, powered to obtain significant results within subgroups of time since last screen.

## Methods

### Study design and participants

This open, two-arm, randomised controlled trial was done in six screening sites in England (Derby, Hull, Plymouth, Sheffield, southeast London, and west London) for different time lengths between June 2, 2014, and Sept 30, 2015. We chose these sites because we already had links in terms of research on and evaluation of the screening programme. Our prior constraints were that we wanted substantial numbers both within and outside London and areas of varying socioeconomic status. We excluded three screening centres where we were already conducting another trial of pre-appointment reminders. The protocol is available in the appendix (pp 4–20).

Women were invited to breast screening in batches of varying size but typically several hundreds. The date a batch was set up (ie, the list of women to be invited was compiled), was defined as the date the screening episode was opened for each woman in that list. For an individual woman, her screening episode is closed when she attends for screening or after 180 days if she does not attend. Batches of women invited to routine breast cancer screening were randomly assigned (1:1) to be sent either a second appointment with a fixed date and time (intervention) or an open invitation (control) in the event of non-attendance at the first offered appointment. After randomisation, the analysis was restricted to women aged 50–70 years. Women who self-referred for screening, women on an early recall protocol, and women who were invited because of a high risk of breast cancer were not randomised. Some women had more than one recorded invitation in the study period, which resulted in multiple records in the dataset. For these women, the first invitation date was used for reference and participation was based on the first attendance date (if any)—ie, only one of the multiple records was kept. Other exclusions that were judged to be necessary were women invited to screening outside the study period of the screening sites; observations with previous screening appointment more recent than the first offered appointment or date of the screening episode being opened; and observations with a date on the invitation letter for a previous round of screening more recent than the date on the current invitation letter. We also excluded women who participated but had missing dates of attendance, because in those cases it was not possible to determine whether they had attended within 90 days of their first offered appointment or within 180 days of their episode being opened (or neither).

Women were not informed of the study or asked to give consent for three reasons. First, the intervention was a minor variation in invitation practice, which was already standard procedure in some areas of England. Second, previous notification of a possible variation to the second letter of invitation might change the women's behaviour and defeat the purpose of the study. Finally, limiting the study to those interested in participating would render the results non-generalisable. The study was approved by the London Bloomsbury Research Ethics Committee.

### Randomisation and masking

Within each screening centre, every invited woman was allocated a unique number, known as the SX number. At the beginning of the study, a coin toss by the chief investigator (SWD) decided that women with an odd SX number would be allocated to the intervention group (second timed appointment), whereas women with an even SX number would be allocated to the control group (open second invitation). This is a pseudorandomisation approach. Thus, there was no masking; however, the endpoint required no subjective judgment. Women who received the wrong intervention for their group assignment (eg, women randomly assigned to the control group who received a second timed appointment letter by administrative error) were marked with an error code but were still included in analysis.

### Procedures

Women who did not attend their first offered appointment were flagged as such by staff at that clinic on the National Breast Screening System (NBSS). Subsequently, administration staff identified the non-attenders in NBSS who were sent a second invitation within 2 weeks of the non-attended first appointment.

The intervention consisted of an invitation to a second appointment with fixed date and time. The control group received a second invitation that consisted of a letter with a telephone number that the women should call to rebook the missed screening appointment. Differences between the two letters were kept to a minimum so that women receiving second timed appointment letters did not feel pressurised into attending their screening appointment if they had made the decision not to participate. Second timed appointments had to be allocated to non-attenders within 90 days of the missed appointment.

Because many practices already used second timed appointments for non-attenders of breast cancer screening, this study merely represented a minor variation in routine practice. Data were pseudonymised, removing identifying data items such as month and day of birth and postcode, before being sent to the Centre for Cancer Prevention (Wolfson Institute of Preventive Medicine, Queen Mary University of London), where analyses were done.

### Outcomes

The primary endpoint was participation (ie, attendance) within 90 days of the first offered appointment. The key secondary endpoint was participation within 180 days of the screening episode being opened, used formally in the programme as a measure for calculating participation rates (usually referred to as uptake in reports). The endpoint was determined locally and objectively as whether or not the invitee attended for screening. Both endpoints were assessed once data collection was completed on March 1, 2016. Other secondary endpoints were subgroup analyses by prevalent (first) or incident (subsequent) screen status, by time since last attended screen, and by index of multiple deprivation and age. An economic analysis is also planned, but as a separate exercise, since this analysis will be a major analytic effort.

### Statistical analysis

On the assumption that 40% of women who received their first invitation letter would not attend their screening appointment, we required 90% power for a difference of 20% (intervention group) versus 15% (control group) of those re-invited participating within 90 days. We also assumed that 20% of invitees would be non-attenders at their last routine screening; that 15% would not have attended for three screening episodes or more; and that 10% would not have attended for four episodes or more. These proportions were approximations derived from Offman and colleagues' study^8^ and from the 10% never-attenders observed in the West Midlands screening histories project.^9^ We required 80% power in all the subgroups for a difference of 14% (intervention) versus 10% (control) in non-attenders at their last routine screening, of 10% versus 7% in those who had not attended for three screening episodes or more, and of 2% versus 1% in those who had not attended for four screening episodes or more. These proportions corresponded to requirements of 1252, 1085, 1422, and 2515 individuals per group in each of the four categories, respectively. Thus, we required at least 10 060 non-attenders per group in total (corresponding to approximately 50 300 women invited to first screening appointment in the two groups). We asked participating centres to recruit substantially more women than this number as a failsafe measure.

The difference in participation between the two groups was compared with Poisson regression for the primary and key secondary endpoints, offset by the total numbers of invitees. This analysis yielded relative risks (RRs) and 95% CIs for participation, and likelihood ratio tests for significance. We also did prespecified subgroup analyses by prevalent or incident screen status, by time since last attended screen, and by the 2010 Index of Multiple Deprivation (IMD, based on a woman's postcode).^10^ National quintiles of IMD were used. Formal tests for heterogeneity of the effect of the intervention by prevalent or incident status or by socioeconomic status on attendance were also done. Primary analysis and subgroup analyses were by intention to treat, so that women who received the wrong type of letter for their trial group were retained in the analysis as if they had received the correct letter. Other women who were potentially excludable but were kept in the intention-to-treat analysis were those who were being screened at the time of extraction of the dataset, who were permanently or temporarily under care, who died, who moved away, who were not known at their recorded address, who attended for screening but for some reason were not screened, who had been recently screened, or whose reason for attendance or non-attendance was missing or coded as “other”. These potentially excludable women were more likely to be identified in the intervention group than in the control group, since the offer of a timed appointment was more likely to prompt the invitee to inform the service that she had moved away or had already been recently screened. We also estimated the effect of the intervention in each site separately, as a post-hoc exploratory analysis. All analyses were done with Stata/IC version 13.1. This trial is registered with Barts Health, number 009304QM.

### Role of the funding source

The Department of Health Policy Research Programme was given the opportunity to comment on this report before submission for publication. One author (JP) was employed by the funding source but only before the results became available to the authors. The sponsors and funders of the study had no role in data collection, data analysis, data interpretation, study design, or writing of the report. The corresponding author had full access to all the data in the study and had final responsibility for the decision to submit for publication.

## Results

The dataset received from the six screening centres had 33 146 records of women invited for screening at different times between June 2, 2014, and Sept 30, 2015, who did not attend their first appointment (see appendix p 3 for details of recruitment by centre). Women were followed up for more than 180 days after their episode in the current round of screening was opened, and the trial was ended when we estimated that the number of women recruited was larger than the one required by our power calculations. 7092 (21%) of the 33 146 records were excluded after randomisation because of the reasons stated in the Methods—eg, ineligible age or date of invitation, missing attendance record, or multiple records for the same woman (3520 in the intervention group *vs* 3572 in the control group; figure). Of the remaining 26 054 women, 12 807 (49%) had been randomly assigned to receive the intervention of a second timed appointment letter, and 13 247 (51%) to receive an open invitation letter. Characteristics of women included in the analysis were similar between groups (table 1). Most women in both groups were younger than 60 years of age and came from more deprived socioeconomic areas (IMD quintiles 1 and 2; table 1).

**Figure:**
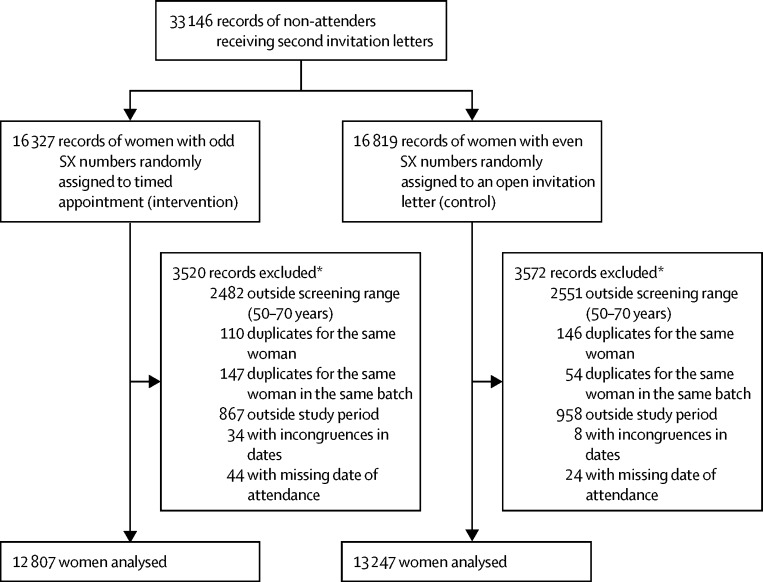
Trial profile SX=a sequential unique identifier of each woman within the NHS Breast Screening Programme. *Some records had more than one reason for exclusion.

**Table 1:** Characteristics of study groups

	**Intervention group (n=12 807)**	**Control group (n=13 247)**
**Screen status**		
Prevalent	6300 (49%)	6517 (49%)
Incident	6507 (51%)	6730 (51%)
**Age (years)**		
50–59	7484 (58%)	7842 (59%)
60–70	5323 (42%)	5405 (41%)
Median (IQR)	58 (53–64)	58 (53–63)
IMD quintile*		
1	3395 (27%)	3623 (27%)
2	3645 (28%)	3703 (28%)
3	2864 (22%)	2978 (22%)
4	1946 (15%)	2028 (15%)
5	939 (7%)	891 (7%)
Missing	18 (<1%)	24 (<1%)
**Site**		
Derby	2398 (19%)	2355 (18%)
Hull	2120 (17%)	2233 (17%)
Plymouth	2456 (19%)	2611 (20%)
Southeast London	1595 (12%)	1638 (12%)
Sheffield	1207 (9%)	1185 (9%)
West London	3031 (24%)	3225 (24%)

Data are n (%) unless otherwise stated.

* 2010 Index of Multiple Deprivation (IMD) from most deprived to most affluent.

586 (5%) of 12 807 women in the intervention group and 52 (<1%) of 13 247 women in the control group received the incorrect invitation letter for their group but were still included in the intention-to-treat analysis as if they had received the correct letter. 455 (4%) women in the intervention group and 119 (<1%) women in the control group were judged to be potentially excludable, because of the nature of the intervention (as noted in the Methods section). The majority of these women were either recently screened and so invited in error (121 [21%] of 574), had missing reason for non-attendance (162 [28%]), or were not known to be or no longer living at the address held (194 [34%]). These women were also retained in our intention-to-treat analyses.

In total, 4493 (17%) of 26 054 women participated in breast cancer screening within 90 days of the date of their first offered appointment. A significantly higher proportion of women in the intervention group participated within 90 days than did women in the control group (2861 [22%] of 12 807 *vs* 1632 [12%] of 13 247; RR 1·81 [95% CI 1·70–1·93], p<0·0001; table 2). A higher proportion of women in the intervention group than in the control group also met the secondary endpoint of participation within 180 days of the screening episode being opened (RR 1·77 [95% CI 1·67–1·88], p<0·0001). Similar results were obtained in a post-hoc per-protocol analysis and an analysis excluding the women deemed potentially excludable (data not shown).

**Table 2:** Participation at second invitation for all women in the trial

	**Intervention group (n=12** **807)**	**Control group (n=13** **247)**	**Absolute difference in attendance**	**RR (95% CI)**	**p value**
Within 90 days of first offered appointment	2861 (22%)	1632 (12%)	10%	1·81 (1·70–1·93)	<0·0001
Within 180 days of episode opened	3054 (24%)	1784 (13%)	10%	1·77 (1·67–1·88)	<0·0001

Data are number who participated (%) unless otherwise stated. RR=relative risk. Percentages have been rounded up.

Overall, 12 817 (49%) of 26 054 women were offered a prevalent screen (6300 in the intervention group *vs* 6517 in the control group) and 13 237 (51%) women were offered an incident screen (6507 in the intervention group *vs* 6730 in the control group). To take into account the fact that the prevalent screen includes women who have been invited before but have never attended, we first analysed younger women in the prevalent round (ie, those aged 50–52 years). In this subgroup, participation within 90 days of the first offered appointment was significantly greater for women in the intervention group than in the control group (table 3); participation within 180 days of the episode being opened supported this result. Next, we analysed data for prevalent screen women aged 53–70 years, who had never previously attended. Although numbers were small, participation at second invitation was still significantly higher in the intervention group than in the control group (table 3).

**Table 3:** Participation at second invitation for prevalent screen women, by age group

		**Intervention group**	**Control group**	**Absolute difference in attendance**	**RR (95% CI)**	**p value**
**50–52 years**						
Number invited		2017	2072	..	..	..
Participation in screening						
	Within 90 days of first offered appointment	347 (17%)	147 (7%)	10%	2·42 (1·99–2·95)	<0·0001
	Within 180 days of episode opened	369 (18%)	163 (8%)	10%	2·33 (1·93–2·80)	<0·0001
**53–70 years**						
Number invited		4283	4445	..	..	..
Participation in screening						
	Within 90 days of first offered appointment	283 (7%)	82 (2%)	5%	3·58 (2·80–4·58)	<0·0001
	Within 180 days of episode opened	307 (7%)	97 (2%)	5%	3·28 (2·61–4·13)	<0·0001

Data are number who participated (%) unless otherwise stated. RR=relative risk. Percentages for the difference in attendance have been rounded up.

Participation data for incident screen women aged 53–70 years who had attended any time previously are shown in table 4 by time since last attended screen before the date of the first offered appointment for this screening episode. Age intervals were adjusted accordingly (eg, only women aged 56–70 years were included in the group who last attended their screening 6–9 years before their first offered appointment). Despite numbers of women participating diminishing with increasing time since last attendance, all results were significantly in favour of the intervention, even for women who had attended previously but 9 years or more before their first offered appointment (table 4). For women who had last attended 1–3 years previously, the expected proportion of women participating in screening within 180 days in the intervention group if there had been no effect of the intervention is 32% (attendance in the control group) of 2853 (number invited in the intervention group, n=912). Therefore, the effect of 2853 second timed appointments was generating 495 (ie, 1407 [the number of attendees in the intervention group]–912 [the number expected of attendees]) attended screens. Thus, in this group about six (2853/495) second timed appointments would have to be offered per additional participant attending screening. The corresponding numbers of second timed appointments required per additional participant are six, 15, and 26 for women whose last attendance was 3–6 years, 6–9 years, and 9 or more years before their current first offered appointment, respectively, calculated as for those attending 1–3 years previously.

**Table 4:** Participation at second invitation for incident screen women, overall and by time since last attendance

		**Intervention group**	**Control group**	**Absolute difference in attendance**	**RR (95% CI)**	**p value**
**Attended any time previously**						
Number invited		6507	6730	..	..	..
Participation in screening						
	Within 90 days of first offered appointment	2231 (34%)	1403 (21%)	13%	1·64 (1·53–1·78)	<0·0001
	Within 180 days of episode opened	2378 (37%)	1524 (23%)	14%	1·61 (1·51–1·73)	<0·0001
**Aged 51–70 years who attended 1 to <3 years previously**						
Number invited		2853	2992	..	..	..
Participation in screening						
	Within 90 days of first offered appointment	1307 (46%)	876 (29%)	17%	1·56 (1·43–1·71)	<0·0001
	Within 180 days of episode opened	1407 (49%)	956 (32%)	17%	1·54 (1·42–1·68)	<0·0001
**Aged 53–70 years who attended 3 to <6 years previously**						
Number invited		1633	1638	..	..	..
Participation in screening						
	Within 90 days of first offered appointment	568 (35%)	306 (19%)	16%	1·86 (1·62–2·14)	<0·0001
	Within 180 days of episode opened	590 (36%)	327 (20%)	16%	1·81 (1·58–2·08)	<0·0001
**Aged 56–70 years who attended 6 to <9 years previously**						
Number invited		529	582	..	..	..
Participation in screening						
	Within 90 days of first offered appointment	71 (13%)	39 (7%)	7%	2·00 (1·35–2·97)	<0·0001
	Within 180 days of episode opened	76 (14%)	45 (8%)	7%	1·86 (1·28–2·69)	<0·0001
**Aged 59–70 years who attended ≥9 years previously**						
Number invited		471	453	..	..	..
Participation in screening						
	Within 90 days of first offered appointment	35 (7%)	16 (4%)	4%	2·10 (1·16–3·81)	0·01
	Within 180 days of episode opened	37 (8%)	18 (4%)	4%	1·98 (1·12–3·48)	0·02

Data are number who participated (%) unless otherwise stated. RR=relative risk. Percentages for the difference in attendance have been rounded up.

Formal tests for heterogeneity of the effect of the intervention by prevalent or incident status were significant (p<0·0001 for participation within 90 days of the first offered appointment and within 180 days of the episode being opened). Separate results for prevalent and incident screens can be seen in Table 3, Table 4. Although the relative effects are larger in the prevalent screen women, the absolute differences in participation are larger in the incident screen women (Table 3, Table 4).

Results did not vary substantially by age group (data not shown). Results by national IMD quintile are shown in table 5. We excluded 42 women who had missing IMD data from this analysis (18 in the intervention group and 24 in the control group). The first two quintiles (1 and 2), corresponding to the most deprived populations, have higher RRs than the other quintiles for participation within 90 days of the first offered appointment and participation within 180 days of the episode being opened. From the third to fifth quintiles, RRs decrease for more affluent women. However, it should be noted that the absolute differences in participation between the two groups were similar, at around 10%, in all quintiles. Results were highly significant in all quintiles (p<0·0001 in all cases).

**Table 5:** Participation at second invitation for all national IMD quintiles (from most to least deprived)

		**Intervention group**	**Control group**	**Absolute difference in attendance**	**RR (95% CI)**	**p value**
**IMD quintile 1 (most deprived)**						
Number invited		3395	3623	..	..	..
Participation in screening						
	Within 90 days of first offered appointment	639 (19%)	353 (10%)	9%	1·93 (1·69–2·20)	<0·0001
	Within 180 days of episode opened	682 (20%)	386 (11%)	9%	1·89 (1·66–2·14)	<0·0001
**IMD quintile 2**						
Number invited		3645	3703	..	..	..
Participation in screening						
	Within 90 days of first offered appointment	768 (21%)	398 (11%)	10%	1·96 (1·73–2·22)	<0·0001
	Within 180 days of episode opened	825 (23%)	434 (12%)	11%	1·93 (1·71–2·17)	<0·0001
**IMD quintile 3**						
Number invited		2864	2978	..	..	..
Participation in screening						
	Within 90 days of first offered appointment	686 (24%)	402 (13%)	10%	1·77 (1·56–2·01)	<0·0001
	Within 180 days of episode opened	734 (26%)	442 (15%)	11%	1·73 (1·53–1·95)	<0·0001
**IMD quintile 4**						
Number invited		1946	2028	..	..	..
Participation in screening						
	Within 90 days of first offered appointment	488 (25%)	297 (15%)	10%	1·71 (1·48–1·98)	<0·0001
	Within 180 days of episode opened	519 (27%)	324 (16%)	11%	1·67 (1·45–1·92)	<0·0001
**IMD quintile 5 (least deprived)**						
Number invited		939	891	..	..	..
Participation in screening						
	Within 90 days of first offered appointment	277 (29%)	178 (20%)	10%	1·48 (1·22–1·79)	<0·0001
	Within 180 days of episode opened	290 (31%)	194 (22%)	9%	1·42 (1·18–1·71)	<0·0001

Data are number who participated (%) unless otherwise stated. IMD=2010 Index of Multiple Deprivation. RR=relative risk. Percentages fhave been rounded up.

In our post-hoc exploratory analyses, the intervention significantly increased participation at all study sites (p<0·0001 in all centres; appendix p 2) compared with that in the control group. The effect of the intervention was highest in southeast London (RR for participation within 90 days of the first offered appointment 2·27 [95% CI 1·90–2·72]), which had the highest proportion of women in the two most deprived IMD quintiles (3249 [87%] of 3738), and only 53 (1%) women in the two most affluent quintiles. By contrast, the effect of the intervention was smallest in Plymouth (RR 1·55 [95% CI 1·36–1·77]), where 3595 (50%) of 7190 women were in the two most deprived IMD quintiles and 1706 (24%) were in the two most affluent quintiles.

## Discussion

In this study, the intervention of inviting non-attenders of breast cancer screening to a second appointment with a fixed date and time caused an absolute increase in participation of 10·3% compared with an open invitation, which would translate to an increase in participation of 3%, since around 30% of invitees to a first appointment were non-attenders.^1^ Most women included in our analysis were younger than 60 years of age and came from more deprived socioeconomic areas.

A limitation of this study was that more than 20% of women were excluded after randomisation. Most excluded women were outside the screening age range, so this factor is unlikely to be a source of bias. Moreover, although the six sites were spread across England, they might not be representative of the English population overall. However, we have no reason to believe that this is the case, and the primary result was similar in all centres. Also, we used pseudorandomisation, allocating to trial group by whether the SX number was odd or even. However, because SX numbers are not assigned in a systematic way by screening centres, the practice should allow valid comparison of trial groups. The control group was larger than the intervention group—ie, there were more women with even SX numbers. Since the SX numbers used for allocation in this trial are assigned strictly sequentially at first invitation to the programme, we are unsure why this difference occurred. We have been unable to identify a systematic factor or staff action that could have caused this imbalance, although centres with more error codes for the sending of the wrong letter were also those with the larger imbalances between the sizes of the two groups. However, analysis was by intention to treat. Notably, the previous trial of second timed appointments for non-attenders had a similar imbalance, although in the other direction (more odd than even SX numbers).^6^

Nearly twice as many women in the intervention group than the control group participated in screening at second invitation. Although the intention-to-treat analysis led to more diluted results than less statistically cautious approaches, the improvement in participation in the intervention group was still substantial, as shown by our primary analysis. These results, therefore, support the NHSBSP policy of second timed appointments. The greater effect at prevalent screen than incident screen is important, since a stronger decline in participation over time has been reported at first invitation than at subsequent invitations.^1^ The universal adoption of second timed appointments for non-attenders at first invitation could go some way to rectifying this trend.

Evidence at the ecological and individual level from Europe and North America suggests that socioeconomic deprivation, often in conjunction with specific ethnicity, is strongly associated with non-participation in breast cancer screening.^11^, ^12^, ^13^, ^14^, ^15^, ^16^ Transport issues and difficulties in getting to the screening appointment are cited as reasons for non-participation.^12^, ^14^ Further detailed surveys of non-participants are needed to improve our understanding of the barriers to participation. In our trial, women living in areas of higher deprivation showed a better response to the intervention than did women living in areas of lower deprivation. Thus, the practice of second timed appointments for non-attenders could address in part the socioeconomic gradient in delivery of the breast screening service.^2^, ^3^ At the very least, this practice should not exacerbate the problem. This gradient needs to be addressed because the clinical stage of presentation tends to be later for women of lower socioeconomic status than for women of higher status, and is a factor that contributes to worse treatment outcomes.^11^ Arguably, a key factor responsible for women not attending their breast screening invitation might be car ownership, which is strongly correlated with socioeconomic status and positively associated with breast screening coverage.^12^ A comparison of car ownership within the same IMD quintile between women who responded to their second timed appointment letter and women who did not could be interesting.

The greatest effect of the intervention was seen in southeast London, which had the highest proportion of women in the two lowest IMD quintiles. Plymouth, with the second largest proportion of study participants in the two lowest IMD quintiles, showed the smallest (albeit still substantial) effect of the intervention. In view of our findings, and the internationally observed socioeconomic gradient in participation in breast cancer screening,^14^, ^15^, ^16^ our results could have relevance to other countries with organised screening programmes.

In all time-interval categories into which women were divided, the proportions of women who participated in screening increased in the intervention group versus the control by around the same relative factor for second timed appointments and there did not seem to be a trend between time since last screen and efficacy of the intervention, by contrast with findings reported by Stead and colleagues.^6^ However, the absolute effect declined with time since last attended screen, as has been reported previously, and reflecting generally similar or larger relative effects but smaller absolute effects in those with a lower baseline participation. The number of second timed appointment letters that need to be offered per additional participant increases for women whose last attendance at breast cancer screening was more than 6 years before their current first offered appointment. Of course, fewer second timed appointment letters have to be issued than open letters per participant; however, reservation of the appointment time is the call on resources.

The economic implications of compulsorily extending the intervention to all women in the breast cancer screening programme will have to be examined before such a change in policy is made, because allocating time slots for fixed timed appointments has a cost in terms of resources. In most cases, overbooking for screening appointments is advised to minimise the waste from unused slots; our results suggest that increasing the overbooking ratio for second timed appointments for previous non-attenders would be safe. However, with the higher variability of the likelihood of participation in the second timed appointments, it would be prudent to mix small numbers of these within sessions with a majority of first offered appointments. Booking software (eg, Smart Clinics) is now available that overbooks by a magnitude depending on the likelihood of attendance. Offering second timed appointments only to those who have attended in the 6 years previous to their first offered appointment might be a cost-effective approach, since it leads to fewer wasted appointments than in those who have not attended for a longer period. This strategy, however, raises questions of ethics and equity and should be considered further by the Department of Health and Public Health England to determine the appropriate policy for the programme. Scope also exists for qualitative research into the public acceptability of the policy of second timed appointments.

In other countries, such as Italy,^17^, ^18^ fixed appointments rather than open invitations have been associated with improved participation in screening in general, not only for non-attenders. Other methods that can successfully increase participation in breast cancer screening are text message,^19^, ^20^ postal,^21^ and telephone reminders;^22^ general practitioner endorsement;^23^, ^24^ and the possibility to change appointments to out-of-office hours.^8^ Second timed appointments for non-attenders could be implemented with these methods (eg, with the second timed appointment letter offering the opportunity to change to an out-of-hours time slot, or having primary care endorsement) and other interventions to improve delivery to all eligible women, with the ultimate goal of improving early detection of breast cancer in England and worldwide.
