# Supplementary material for: Effect of second timed appointments for non-attenders of breast cancer screening in England: a randomised controlled trial
Source: Lancet Oncol. 2017 Jul;18(7):972–80. doi: 10.1016/S1470-2045(17)30340-6 (PMC5489696; doi:10.1016/S1470-2045(17)30340-6)
Supplement: Supplementary appendix [file mmc1.pdf]

THE LANCET
Oncology

Supplementary appendix

This appendix formed part of the original submission and has been peer reviewed. We post it as supplied by the authors.

Supplement to: Allgood PC, Maroni R, Hudson S, et al. Effect of second timed appointments for non-attenders of breast cancer screening in England: a randomised controlled trial. Lancet Oncol 2017; published online May 15. http://dx.doi.org/10.1016/S1470-2045(17)30340-6.

THE EFFECT OF SECOND TIMED APPOINTMENTS FOR NON-ATTENDERS OF BREAST CANCER SCREENING IN ENGLAND: A RANDOMISED CONTROLLED TRIAL

PC Allgood, R Maroni, S Hudson, et al

Web Appendices

Appendix A.

Table 6. Participation (at second invitation) by randomisation for each screening site separately.

	Intervention group	Control group	RR (95% CI)	p-value	Absolute increase in attendance in the intervention group
**TOTAL INVITED**	**2,398**	**2,355**			
**Derby**					
Within 90 days of first offered appointment	678 (28·3%)	406 (17·2%)	1·64 (1·45-1·86)	< 0·0001	+ 11·0%
Within 180 days of episode opened	688 (28·7%)	417 (17·7%)	1·62 (1·43-1·83)	< 0·0001	+ 11·0%
**TOTAL INVITED**	**2,120**	**2,233**			
**Hull**					
Within 90 days of first offered appointment	372 (17·5%)	227 (10·2%)	1·73 (1·46-2·04)	< 0·0001	+ 7·4%
Within 180 days of episode opened	426 (20·1%)	252 (11·3%)	1·78 (1·52-2·09)	< 0·0001	+ 8·8%
**TOTAL INVITED**	**2,456**	**2,611**			
**Plymouth**					
Within 90 days of first offered appointment	568 (23·1%)	389 (14·9%)	1·55 (1·36-1·77)	< 0·0001	+ 8·2%
Within 180 days of episode opened	593 (24·1%)	404 (15·5%)	1·56 (1·37-1·78)	< 0·0001	+ 8·7%
**TOTAL INVITED**	**1,595**	**1,638**			
**South East London**					
Within 90 days of first offered appointment	394 (24·7%)	178 (10·9%)	2·27 (1·90-2·72)	< 0·0001	+ 13·8%
Within 180 days of episode opened	426 (26·7%)	205 (12·5%)	2·13 (1·80-2·53)	< 0·0001	+ 14·2%
**TOTAL INVITED**	**1,207**	**1,185**			
**Sheffield**					
Within 90 days of first offered appointment	306 (25·4%)	164 (13·8%)	1·83 (1·51-2·22)	< 0·0001	+ 11·5%
Within 180 days of episode opened	330 (27·3%)	183 (15·4%)	1·77 (1·47-2·13)	< 0·0001	+ 11·9%
**TOTAL INVITED**	**3,031**	**3,225**			
**West London**					
Within 90 days of first offered appointment	543 (17·9%)	268 (8·3%)	2·16 (1·86-2·50)	< 0·0001	+ 9·6%
Within 180 days of episode opened	591 (19·5%)	323 (10·0%)	1·95 (1·69-2·23)	< 0·0001	+ 9·5%

Appendix B. Recruitment by site.

Site	Subjects pre-exclusions	Subjects post-exclusions	Principal investigator	Period of enrolment of subjects
West London	8,124	6,256	Julie Somers	02/06/2014 – 14/01/2015
Plymouth	7,199	5,067	Jim Steel	29/08/2014 – 30/09/2015
Derby	6,145	4,753	Anne E. Turnbull	04/11/2014 – 27/08/2015
Hull	4,973	4,353	Lesley Peacock	27/10/2014 – 14/04/2015
South-East London	3,749	3,233	Geraldine Kirby	26/08/2014 – 16/12/2014
Sheffield	2,956	2,392	Christine E. Ingram	14/07/2014 – 31/12/2014

Appendix C. Study Protocol

STUDY PROTOCOL

Version 0.8

A randomised controlled trial of the effect of second timed appointments for non-attenders for breast cancer screening on population uptake

Short title/Acronym: Second Timed Appointment Study

Sponsor: Queen Mary, University of London

Representative of the Sponsor:

Gerry Leonard

Head of Resources

Joint Research Mangement Office

5 Walden Street

London

E1 2EF

Phone: 020 7882 7260

Email: sponsorsrep@bartshealth.nhs.uk

REC reference: Insert once known

CHIEF INVESTIGATOR AGREEMENT PAGE

The clinical study as detailed within this research protocol (Version XXX, dated XX XXX XX), or any subsequent amendments will be conducted in accordance with the Research Governance Framework for Health & Social Care (2005), the World Medical Association Declaration of Helsinki (1996) and the current applicable regulatory requirements and any subsequent amendments of the appropriate regulations.

Chief Investigator Name: **Prof. Stephen W. Duffy**

Chief Investigator Site: **QMUL**

Signature and Date:

29th October 2013

STUDY SUMMARY/SYNOPSIS

**TITLE**	A randomised controlled trial of the effect of second timed appointments for non-attenders for breast cancer screening on population uptake
**SHORT TITLE**	Second Timed Appointment Study
**Protocol Version Number and Date**	Version: 0.8
**Methodology**	Randomised Controlled Study
**Study Duration**	1 November 2013 to 30 November 2014
**Study Centre**	Centre for Cancer Prevention, Wolfson Institute, Queen Mary, University of London (QMUL)
**Objectives**	To estimate the difference in attendance rate between non-attenders given a second timed appointment and those given an open letter asking the invitee to telephone to make a second appointment.
**Number of Subjects/Patients**	50,300 women
**Main Inclusion Criteria**	Women in consecutive GP batches to be invited for routine breast screening appointments.
**Statistical Methodology and Analysis**	Overall attendance in the two arms will be compared using logistic regression and subgroup analyses will be performed for the various categories of non-attenders, prevalent and incident rounds, patient age and IMD (index of multiple deprivation).

CONTENTS

1. STUDY INVESTIGATORS 7
2. ABBREVIATIONS 9
3. STUDY SUMMARY 10
3.1. Aim 10
3.2. Study design 10
3.3. Data analysis 10
4. BACKGROUND 11
5. AIM OF THE STUDY 11
6. STUDY DESIGN 12
7. SUBJECT SELECTION 13
7.1. Inclusion 13
7.2. Exclusion 13
8. DATA HANDLING AND RECORD KEEPING 13
9. DATA ANALYSIS 14
9.1. Attendance analysis 14
9.2 Power and sample size 11
9.3. Economic analysis 15
10. ETHICAL CONSIDERATIONS 15
11. INFORMATION GOVERNANCE 15
12. FUNDING 16
13. REPORTING AND DISSEMINATION 16
14. REFERENCES 18
15. APPENDICES 19

STUDY INVESTIGATORS

Dr Anthony Maxwell
Consultant Radiologist and North West Regional Director of Breast Screening Quality Assurance

Royal Bolton Hospital
Minerva Road
Bolton BL4 0JR
E-mail: anthony.maxwell@nhs.net
Tel: 01204 390046

Dr Anthony Threlfall
Independent Research Consultant,
134 Slade Lane
Manchester M19 2AQ
E-mail: anthony.threlfall@googlemail.com
Tel: 0161 212 6095

Professor Stephen Duffy
Professor of Cancer Screening
Centre for Cancer Prevention
Wolfson Institute of Preventive Medicine
Barts and The London
Charterhouse Square
London EC1M 6BQ
E-mail: s.w.duffy@qmul.ac.uk
Tel: 020 7882 3535

Dr Rhian Gabe
Senior Lecturer in Clinical Trials
Hull York Medical School
University of York
YO10 5DD
E-mail: rhian.gabe@york.ac.uk
Tel: 01904 321399

Dr Judith Offman
Intervention Research Manager
Breast Cancer Care
5 - 13 Great Suffolk Street
London, SE1 0NS
E-mail: judith.offman@breastcancercare.org.uk
Tel: 020-7960 3447

Dr Prue Allgood
Research Associate
Centre for Cancer Prevention
Wolfson Institute of Preventive Medicine
Barts and The London
Charterhouse Square
London EC1M 6BQ
E-mail: p.allgood@qmul.ac.uk

Tel: 020-7882 3525

ABBREVIATIONS

CCP	Centre for Cancer Prevention
DOBS	Director of Breast Screening
GP	General Practitioner
IMD	Index of multiple deprivation
LSOA	Lower Super Output Area
NBSS	National Breast Screening System
NHS	National Health Service
NHSBSP	National Health Service Breast Screening Programme
NRES	National Research Ethics Service
SOM	Screening Office Manager
SSA	Site Specific Approval
QMUL	Queen Mary, University of London

STUDY SUMMARY

3.1. Aim

To estimate the difference in overall attendance rate between non-attenders given a second timed appointment and those given an open letter asking the invitee to telephone to make a second appointment. In particular, we wish to estimate this difference for various categories of non-attenders.

3.2. Study design

A randomised controlled trial with 1:1 randomisation. Women who have not attended their first timed appointment will be either sent a letter with a second timed appointment, or a letter asking them to telephone to make a second appointment, the latter being routine procedure. The total number of women to be recruited is 50,300 invitees (approximately 20,120 non-attenders at first offered appointment) (10,060 non-attenders in each arm) from six screening services, two in London, two in Yorkshire, one in Derbyshire and one in the south west.

3.3. Data analysis

The study endpoints will be whether or not the subject attends for screening within 90 days of the first offered appointment and within 180 days of the episode being opened, the latter being the formal definition of uptake within the screening programme. In addition, subgroup analyses looking at previous screening history, socio-economic status and age will be performed.

It will also be of some interest to observe whether the results for the London units differ from those outside the capital, as uptake in London is further complicated by population mobility, which can mean invitees changing addresses between initial invitation and second contact following non-attendance [1].

In addition to the efficacy of the strategy in terms of uptake, the resource implications of unattended second timed appointments will also be estimated.

BACKGROUND

Reduction in breast cancer mortality in the UK depends in part upon maximising the number of women who attend for routine breast screening. The service has a duty to allow the women invited to make up their own mind as to whether to take up the offer of screening, but it would be unethical not to acknowledge that the service is offered because screening has been found to prevent deaths from breast cancer. Attendance rates for screening have slowly declined nationally over the last few years. In 2004-5 74.6% of invited women aged 50-70 attended, whereas that had fallen to 73.5% in 2009-10. The fall is more marked for women in the first (prevalent) round of screening, where attendance across the UK fell to 69.7% in 2009-10, compared with 71.0% in 2005-6, and is now below the national minimum standard of 70% (2, 3).

Policy on sending second appointments to those who do not attend the first appointment offered has varied amongst screening units. Until recently, most supplied open invitations, inviting the non-attender to make an alternative appointment, whereas in some areas, for example some London PCTs, second timed appointments have been offered routinely. One trial [4] in the 1990's found an increased rate of attendance with second timed appointments as opposed to 'open' letters inviting the women to telephone to make a second appointment (23% vs 12%, p<0.001). This would correspond to a 3% increase in the overall participation rate. A more recent study in North London (Brazil, personal communication) also achieved a 3% overall improvement.

The practice of offering second timed appointments is a significant call on resources, particularly when applied to persistent non-attenders, a population with a high probability of not responding. In addition, much has changed since the early years of the breast screening programme, and it is not clear that the results of Stead et al's trial [4] would still apply today.

While practice varies among units, appointment non-attenders are usually processed one clinic at a time, either before the results for the women who attended are ready or after all the mammograms for a clinic have been read, depending on screening unit policy. At this point the clinic episodes for all non-attenders are closed and an 'open invite' is issued, which is the default in most screening units. In units that currently invite to 2nd timed appointments screening episodes are left open, the women are allocated a 2nd timed appointment and are sent a new invitation letter. Both closing the screening episodes and allocating second timed appointment letters is carried out manually by screening office staff.

AIM OF THE STUDY

To estimate the difference in attendance rate between non-attenders given a second timed appointment and those given an open letter asking the invitee to telephone to make a second appointment. In particular, we wish to estimate this difference for various categories of non-attenders and socio-economic status.

STUDY DESIGN

This is a randomised controlled trial. At the time when a batch is called up and women are sent an invite for their routine screen, they will also be randomised 1:1 to be sent either an open invitation letter or a second timed appointment in the event of non-attendance of this first appointment. Randomisation will be by each woman's screening reference number (Sx number). At the commencement of the study a simple coin toss will be used to determine whether women with odd or even SX numbers will receive the letter with a second timed appointment or an open appointment letter. There is no reason to believe that a difference exists between the characteristics of women with odd and even Sx numbers.

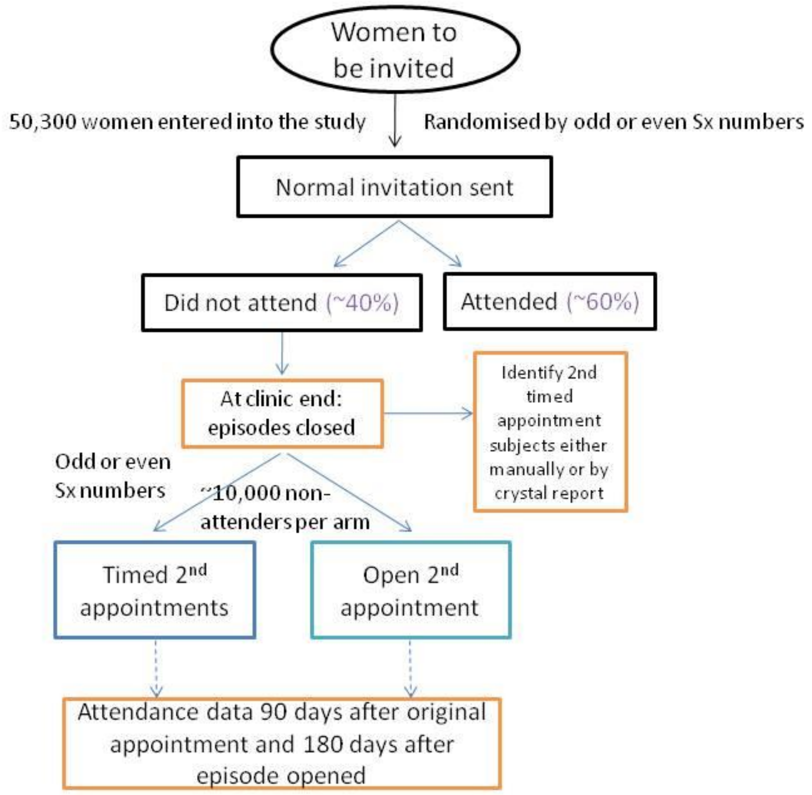

The trial is summarised in Figure 1. Women are called up in batches to be invited for their regular screening appointment, and receive an invitation letter following normal screening unit procedures. Non-attenders will be processed following usual screening unit procedure as described above. A crystal report will be run before a clinic is processed, as an aide-memoire to the staff working on rebooking appointments, and to ensure that no invitee is missed. This report will identify non-attenders who are part of the study and the study arm into which the women had been randomised. Using this list the screening office manager or clerical staff will manually either close the episode or

allocate them a new appointment, depending on the trial arm. Once a clinic has been processed, both open and timed appointment letters will be produced and posted following the screening unit's standard procedures. Second timed appointments should be allocated to non-attenders within 90 days of the missed appointment. A crystal report that analyses the NBSS Letter Log table will be run monthly to confirm that all DNA women in a clinic have had the correct letter. In addition, this extract will be sent to the CCP for monitoring purposes and to assess progress to date.

Second timed appointment letters will be the same as the open invitation letter used at each screening unit but with appointment details replacing the sentence asking women to call in to make an appointment. As this constitutes only a minor variation to routine practice, and second timed appointments are already being used by some breast screening services, women will not be consented before or informed about being entered into the study.

Attendance will be assessed after 90 days of the first offered appointment and 180 days after the episode was opened. Attendance data extracts for all women invited from a batch (including women who attended their first appointment) will be downloaded after 90 days of their first offered appointment and after 180 days of the episode being opened.

The study period will be from the 1st November 2013. We anticipate that seven months of recruitment will deliver the 50,300 invitees. Reporting of results to the National Breast Screening Programme will be done in two stages, in Autumn 2014 on first endpoint data and about 3 months later on second endpoint data. Submission to the peer-reviewed literature will take place once both first and second endpoint data have been analysed.

End of study: Last data set received at CCP.

SUBJECT SELECTION

7.1. Inclusion

Women invited in batches to their routine breast screening appointment.

7.2. Exclusion

Exclusion criteria:

- Self referrals
- Early re-calls
- Women who are invited because of a higher risk of breast cancer

DATA HANDLING AND RECORD KEEPING

Crystal reports will be run before a clinic is processed to identify non-attenders who are part of the study and the study arm into which the women had been randomised. The type of second appointment letter sent is logged on the NBSS system when a clinic is being processed. A Crystal Report that analyses the NBSS Letter Log table will be run monthly and a pseudo-anonymised copy of this data extract sent to the CCP for the study coordinator to monitor that the correct letters were sent.

The attendance details of each woman in the study are recorded on the National Breast Screening System (NBSS) in the same way as for women who are not in the study.

9. DATA ANALYSIS

9.1. Attendance analysis

Two further Crystal reports will extract the attendance data (see appendix) for women in the intervention and control arms, first to include data on all subjects up to at least 90 days after first offered appointment (i.e. at least 90 days after the first offered appointment of the latest recruit to the study), then to include data on all subjects up to at least 180 days after the screening episode opened. Downloaded data from the Crystal reports will be transferred in anonymised form to the Centre for Cancer Prevention (CCP), Queen Mary, University of London, for analysis. The difference in attendance rate between the two arms (at exactly 90 days after each individual first offered appointment and at exactly 180 days after each individual episode opening date) will be compared using logistic regression and subgroup analyses will be performed for those women who have attended in the past, by time since last attendance, those who have been invited and not attended in the past, those invited for the first time, patient age and IMD (index of multiple deprivation). Two endpoints will be used:

Attendance report 1 (primary endpoint):

- Attendance of non-attenders comparing open and second timed appointments within 90 days of the date of first offered appointment

Attendance report 2 (secondary endpoints):

- attendance non-attenders within 180 days of the episode being opened, which is the time frame used to calculate screening uptake
- total uptake of all women invited within 180 days of the episode being opened
- subgroup analyses by prevalent/incident status
- subgroup analysis by non-attendance categories (time since last attended screen)
- subgroup analysis by index of multiple deprivation (IMD) and age

9.2 Power and study size

In terms of sample size, the major objective of the study is to determine whether for some groups (e.g. those who have not attended their last three episodes), the use of second timed appointments makes no worthwhile addition to attendance. We anticipate rather smaller effects than observed by Stead et al [4]. Table 1 shows the required numbers in non-attendance categories and required total numbers of invitees, for various postulated effects. We assume that 40% of invitees would not attend the first offered appointment, that 20% will have been non-attenders at last episode (as evidenced by more than four years since last screen), that 15% will have not attended for three episodes or more (more than 7 years since last screen) and that 10% will not have attended for four episodes or more (more than 10 years since last screen). These figures are conjectured, but are consistent with the 10% never attenders in the West Midlands Screening Histories Project and with results of the Out of Hours Study [5,6]. They are also broadly consistent with findings in a study of second timed appointments in North London in 2010 (Judith Offman, personal communication).

We require 90% power for the main effect and 80% in all subgroups of interest. For a difference of 15% vs 20% of those reinvited attending within 90 days in the 40% anticipated not attending the

first, we would have 90% power for a significant result with 2,504 non attenders, i.e. 6,260 invitees in all. For a difference of 10% vs 14% in women aged 53 or more with more than four years since last attendance , we would need 2,170 women in this category, i.e. 10,850 initial invitees. For a difference of 7% vs 10% in those with seven or more years since last attendance, we would need 2844 women in the category, and 18,960 invitees. For a difference of 1% vs 2% attendance in those with more than ten years, we would need 5,030 women in this category of non-attendance. We would need therefore, 50,300 invitees in all, which would give ample power for all other subgroups of interest. The difference of 1% vs 2% in the latter category (i.e. a difference of only 0.1% to overall attendance) would not be considered worthwhile, but having the power to detect it means that a policy decision as to when it is reasonable to stop offering second timed appointments would be based on very precise information.

9.3. Economic analysis

The cost of each additional attendance in each group will be calculated using the cost of running second timed appointment clinics with potential unfilled appointment slots and the clerical time required to manually book these appointments. Cost calculation will vary with local policy as regards organisation of screening capacity for those who do not attend the first offered appointment.

10. ETHICAL CONSIDERATIONS

As non attenders in the control arm receive the open second screening invitation and women in the intervention arm are only given a timed second appointment, this study merely represents a minor variation on routine practice. Furthermore, second timed appointments are already being employed by some breast screening units in the UK. Women entered into this study will therefore not be informed that they are subjects in a study or asked for consent. In addition, eliciting prior consent to be sent a second timed appointment invitation would result in a serious loss of generalisability. No ethical issues are anticipated in view of the minor nature of the intervention.

To ensure that the women receiving second appointment letters do not feel pressurised into attending their screening appointment if they have made the decision not to attend, second timed appointment invitations are only altered slightly from the standard open invitation letter.

The study protocol will be submitted to the National Research Ethics Service (NRES) to seek favourable approval from a main Research Ethics Committee. Site Specific Approval (SSA) and R&D approval will also be obtained.

INFORMATION GOVERNANCE

NHS information governance requirements will be observed. Data will be transferred between the participating screening units and the CCP in the form of password protected Microsoft Excel spreadsheets using NHS.net accounts. The only identifiers for individual women will be screening number, year of birth and postcode. Postcodes will only be used to link a Lower Super Output Area (LSOA) code to each woman, which is then used to allocate the Index of Multiple Deprivation (IMD). The IMD and not the postcode will be made available to the data analysis team at QMUL. Our institute has obtained a 79% score on the Information Governance toolkit.

FUNDING

NHS Cancer Screening Programmes has agreed to fund the study in its entirety.

REPORTING AND DISSEMINATION

The results of the study will be presented at one or more scientific meetings and submitted for publication in one or more peer-reviewed journals. It will be tabled at an NHSBSP Breast Screening Quality Assurance Directors' meeting and a meeting of the National Screening Committee.

Table 1. sample size required for various categories of non-attender in second timed appointments study, assuming two equal sized trial arms					
Category	Proportion of screenees	Postulated difference in attendance at second invitation	Power required	Number needed in category (both trial arms combined)	Total screenees needed (both trial arms combined)
All non-attenders at first offered date	40%	15% vs 20%	90%	2,504	6,260
More than 4 years since last attendance	20%	10% vs 14%	80%	2,170	10,850
More than 7 years since last attendance	15%	7% vs 10%	80%	2,844	18,960
More than ten years since last attendance	10%	1% vs 2%	80%	5,030	50,300

REFERENCES

1. Millet C, Zelenyanski C, Binysh K, et al. Population mobility: characteristics of people registering with general practices. Public Health 2005; 119: 632-8

2. NHS Cancer Screening Programmes. NHS Breast Screening Programme: Annual Review 2011. Sheffield2011.

3. NHS Cancer Screening Programmes. NHS Breast Screening Programme: Annual Review 2007. Sheffield2007.4.Stead MJ, Wallis MG, Wheaton ME. Improving uptake in non-attenders of breast screening: selective use of second appointment. J Med Screen 1998; 5: 69-72

4. Stead MJ, Wallis MG, Wheaton ME. Improving uptake in non-attenders of breast screening: selective use of second appointment. J Med Screen 1998; 5: 69-72

5. Lawrence G, O'Sullivan E, Kearins O, Tappenden N, Martin K, Wallis M. Screening histories of invasive breast cancers diagnosed 1989-2006 in the West Midlands, UK: variation with time and impact on 10-year survival. J Med Screen. 2009;16(4):186-92.

6. Offman J, Wilson M, Lamont M, et al. A randomised trial of weekend and evening breast screening appointments. Br J Cancer 2013; 109: 597-602

APPENDIX

1. Non-attender extracts:
    - Clinic Code
    - Clinic Date – (Date of Missed Appointment)
    - Sx No.
    - Date First Offered Appt
    - Year Of Birth
    - P/I
    - Date episode opened
    - Batch ID
    -
    - Study Arm = Second Timed Letter or Open Letter
    - Letter ID of letter sent (or blank if no letter sent yet)
    - Date 2nd timed appointment (will be blank for women sent open letters)
    - Clinic code of 2nd Timed appointment (will be blank for women sent open letters)
    - Operator Code (for audit purposes)
    - Log code (for Audit purposes)
    - Error Code = Y/N (if the study arm and letter type conflict)
    - Client Full Name (needed by Office Staff if they are to use this for re-appointing

If this extract is to be sent to the CCP then the final column (Client Full Name) will be deleted prior to sending.

The extract can be run for a single Clinic/Date to be used by the Office staff or For all clinics in a given period - to be used to make a monitoring extract for the CCP.

2. Attendance extracts
    - Sx No.
    - Batch ID
    - Episode ID - stored for technical reasons
    - Date episode opened
    - Year Of Birth
    - Age at DOFOA
    - Postcode
    - Prevalent/Incident
    - Screening Site = Clinic Code of first offered appointment
    - Date First Offered Appt
    - Date 2nd timed Appointment (blank for open letters)
    - Attended appointment in this episode Y/N
    - Attended appointment Date
    - Reason episode closed (e.g. Opted out, DNA, Recently screened etc)
    - Reason episode closed code (e.g. OT, DNA, RS)
    - Previous attended date
    - Last routine invite date
    - Study Arm = Second Timed Letter or Open Letter

- Letter ID of letter sent (or blank if no letter sent)
- Letter Type Sent
- Error Code = Y/N (if the study arm and letter type conflict)

Postcode will be converted to IMD by the informatics staff and then deleted before transfer to the analysis team at CCP.
